# The principle of respect for autonomy – Concordant with the experience of oncology physicians and molecular biologists in their daily work?

**DOI:** 10.1186/1472-6939-9-5

**Published:** 2008-03-26

**Authors:** Mette Ebbesen, Birthe D Pedersen

**Affiliations:** 1 Centre for Bioethics and Nanoethics, University of Aarhus, Aarhus C, Denmark; 2 Visiting Researcher at the Kennedy Institute of Ethics, Georgetown University, Washington DC, USA; 3 Department of Nursing Science, University of Aarhus, Aarhus C, Denmark; 4 Faculty of Health Sciences, University of Aarhus, Aarhus C, Denmark

## Abstract

**Background:**

This article presents results from a qualitative empirical investigation of how Danish oncology physicians and Danish molecular biologists experience the principle of respect for autonomy in their daily work.

**Methods:**

This study is based on 12 semi-structured interviews with three groups of respondents: a group of oncology physicians working in a clinic at a public hospital and two groups of molecular biologists conducting basic research, one group employed at a public university and the other in a private biopharmaceutical company.

**Results:**

We found that that molecular biologists consider the principle of respect for autonomy as a negative obligation, where the informed consent of patients or research subjects should be respected. Furthermore, molecular biologists believe that very sick patients are constraint by the circumstances to a certain choice. However, in contrast to molecular biologists, oncology physicians experience the principle of respect for autonomy as a positive obligation, where the physician in dialogue with the patient performs a medical prognosis based on the patient's wishes and ideas, mutual understanding and respect. Oncology physicians believe that they have a positive obligation to adjust to the level of the patient when providing information making sure that the patient understands. Oncology physicians experience situations where the principle of respect for autonomy does not apply because the patient is in a difficult situation.

**Conclusion:**

In this study we explore the moral views and attitudes of oncology physicians and molecular biologists and compare these views with bioethical theories of the American bioethicists Tom L. Beauchamp & James F. Childress and the Danish philosophers Jakob Rendtorff & Peter Kemp. This study shows that essential parts of the two bioethical theories are reflected in the daily work of Danish oncology physicians and Danish molecular biologists. However, the study also explores dimensions where the theories can be developed further to be concordant with biomedical practice. The hope is that this study enhances the understanding of the principle of respect for autonomy and the way it is practiced.

## Background

This article presents partial findings of the larger research project 'Bioethics in Theory and Practice', where the overall purpose is (1) to investigate ethical reasoning in biomedical practice in Denmark empirically and (2) to show how to integrate empirical research into the formulation of normative ethical principles without losing the normative approach. Specifically, this article explores how Danish oncology physicians and Danish molecular biologists experience the principle of respect for autonomy in their daily work. The fact that molecular biologists investigate DNA and cells in cultures, whereas oncology physicians work with human beings in a doctor-patient relationship might have the effect that molecular biologists and oncology physicians perceive or experience the principle of respect for autonomy of the patient or the research subject in different ways. During their daily work, molecular biologists conduct basic research, they do not perform animal testing or clinical trials. Among others, they use human material such as DNA and cells in cultures, which derives from either patients samples or existing registered material as for instance cell lines. If the material derives from patient samples, a signed informed consent sheet should be stated by the patient. Molecular biologists do not collect the samples themselves and thereby they do not face the research subject or the patient directly. Findings from the larger research project show that in general, molecular biologists experience that in contrast to animal testing and clinical trials, basic research does not cause significant ethical problems as long as a signed informed consent sheet is available from the research subject or the patient. However, they do face environmental risks regarding radioactivity and chemicals, but these are minimal. On the other hand, oncology physicians consult patients suffering from serious cancer in the out-patients' clinic. Results from the larger research project show that in general, oncology physicians working in the clinic experience a closer relationship between their daily work and ethical problems concerning human beings than molecular biologists. Oncology physicians balance the efficiency and the side effects of treatments, as for instance chemo- and radiation therapy. They consider ethical evaluation as part of the daily work discussing how to treat patients in groups and having interdisciplinary seminars. They experience ethical problems having the character of informed consent and risk-benefit analyses. Furthermore, they perform justice considerations: They have limited resources, i.e. few instruments or devices compared to the number of patients suffering from cancer and they want to help as many patients as possible the best way. This article focus on how Danish oncology physicians and Danish molecular biologists experience the principle of respect for autonomy in their daily work.

### Developing a suitable method

Biomedical ethics has to date largely consisted of theoretical research. Although such theoretical reflections make important contributions to the field, empirical researchers regard some of these attempts as remote from biomedical practice [1]. On the other hand, published empirical research on the ethical reasoning of nurses and physicians offers only descriptions of such reasoning. For instance, a study by Udén et al. [2] on the reflections of nurses and physicians in their narratives about ethically difficult care episodes concludes that the ethical thinking of nurses appears to be related to the ethics of care, whereas the ethical thinking of physicians is related to the ethics of justice. Moreover, the study [3] shows that nurses tell their stories within a relationship ethics perspective and that physicians tell their stories within an action ethics perspective. It remains unclear, however, whether nurses *ought *to assume a care or relationship ethics perspective, or if physicians *ought *to take a justice or action ethics perspective. Can the descriptive conclusions of the study have any normative implications for nurses and physicians? For instance, if an empirical study concludes that physicians adhere to a paternalistic doctor-patient relationship in which physicians do not respect the autonomy of the patient, does such a study then imply that physicians *ought *to adhere to such a paternalistic relationship? So the question remains whether there is any relationship between empirical findings and ethical theory about what *principles *(appendix, note 1) we ought to act in accordance with. We believe by taking an approach to bioethics in which empirical research is integrated into the formulation of normative ethical principles, the conclusions of empirical studies may provide health care professionals and researchers with normative principles about how to analyse, reason and act in practice in ethically difficult situations.

Lindseth & Norberg [4] developed a phenomenological hermeneutical method to reveal the morals and the ethical thinking of physicians and nurses based on interviews. According to Lindseth & Norberg [4] this method can be used to elucidate the essential understandable meaning of good and bad as actually lived in human experience. The method was inspired by Husserl's descriptive phenomenology in as much as the aim is to describe the lived experience of the interviewees. It is essential that the researcher has a phenomenological attitude, and sheds all prior personal knowledge to grasp the essential lived experience of the respondents [5]. Furthermore, it is important that the respondents shift to the phenomenological attitude, i.e. refrain from making judgements about the factual. According to Lindseth & Norberg [4]: "The easiest and, so to speak, the natural way of doing this is to narrate from lived experience". The approach is hermeneutical since the task is to understand the experiences expressed in the interview texts. Hermeneutics goes beyond the description of core concepts and essences to look for meanings embedded in life practices. These meanings are not always apparent to the respondents, but can be gleaned from the narratives (the interview texts) they produce [5]. The results of the phenomenological hermeneutical method of Lindseth & Norberg [4] are descriptive in as much as they describe the lived experience of the respondents. In Ebbesen & Pedersen [6] we argue that the phenomenological hermeneutical method can be combined with the moral philosophical method of Wide Reflective Equilibrium (WRE) as a decision procedure for the formulation of normative principles, because WRE is a method or process of deciding what we should think, not merely one of describing what we do think [7]. So to achieve a normative approach, we combine the phenomenological hermeneutical method with the method of WRE.

The method of WRE is based upon the American philosopher John Rawls' theory for developing and justifying principles for a just society. Rawls speaks of a system with three levels: particular moral judgements, first principles, and general convictions. He claims that particular moral judgements are justified by the overall *coherence *(appendix, note 2) of the system and uses the term WRE to describe this state [8]. To achieve WRE, we start with our initial moral judgments. We begin by screening our initial moral judgements to eliminate those in which we have little confidence and those made under circumstances conducive to error. We then search for general moral principles that best account for the remaining considered moral judgements. We may find reason to revise or discard some of our considered moral judgments that conflict with highly plausible moral principles. Rawls imagines that the process of comparing principles with our considered judgments will lead us to go back and forth, sometimes modifying our principles and sometimes our considered moral judgements until the principles match, fit, or are in line with our considered moral judgements and consistency is achieved. Finally, we have to subject the moral principles we arrive at to alternative moral perspectives and the force of various arguments for these. WRE is achieved when our considered judgements match, or are in line with our general principles duly pruned and adjusted. However, this WRE is not necessarily stable. For instance, it may be liable to be upset by particular cases which lead us to revise our judgments or principles [8,9]. Moreover, the notions of 'match', 'fit', 'in line with' and 'consistency' are not well-defined. We understand the terms as meaning that WRE requires logical consistency between considered moral judgements and moral principles. Rawls writes that the justification of ethical principles "is a matter of the mutual support of many considerations, of everything fitting together into one coherent view [9]." Rawls believes that if reasonable principles exist for deciding moral questions "there is a presumption that the principles of a satisfactory explication of the total range of the considered judgments of *competent judges *(appendix, note 3) will at least approximate them [10]."

In the light of an interpretation of the method of WRE as a decision procedure, the purpose of this empirical study is to validate, formulate and justify reasonable moral principles in specific biomedical practice. To make the approach normative, the interview guide was constructed in accordance with the theory of WRE so that the respondents could achieve WRE. For more details regarding the theoretical framework of the project, please see Ebbesen & Pedersen [6].

### Philosophical background

The word autonomy, derived from the Greek *autos *meaning 'self' and *nomos *meaning 'rule', 'governance' or 'law', originally referred to the self-rule or self-governance of independent city states. Autonomy has since been extended to individuals and has acquired meanings as diverse as self-governance, liberty rights, privacy, individual choice and freedom of the will. Clearly autonomy is not a univocal concept and little agreement exits about its nature, scope or strength [11]. In many books on biomedical ethics the principle of respect for autonomy is one among several important moral considerations that has to be evaluated. The theories of the American bioethicists Tom L. Beauchamp & James F. Childress [11] and the Danish philosophers Jakob Rendtorff & Peter Kemp [12] are examples.

The theory of Beauchamp & Childress is one of the most influential bioethical theories in the world. It emphasises that the principle of respect for autonomy is one among four important ethical principles in biomedical ethics. After examining considered moral judgements in biomedicine, Beauchamp & Childress are convinced that the principles of respect for autonomy, nonmaleficence, beneficence and justice are central to and play a vital role in biomedicine [13]. For clarification, we present below a brief formulation of the bioethical principles of Beauchamp & Childress:

#### The Principle of Respect for Autonomy

• As a negative obligation: Autonomous actions should not be subjected to controlling constraints by others

• As a positive obligation: This principle requires respectful treatment in disclosing information, probing for and ensuring understanding and voluntariness, and fostering autonomous decision-making [11].

#### The Principle of Beneficence

• One ought to prevent and remove evil or harm

• One ought to do and promote good

• One ought to weigh and balance the possible goods against the possible harms of an action [11].

#### The Principle of Nonmaleficence

One ought not to inflict evil or harm. Or more specifically: One ought not to hurt other people mentally or physically [11].

#### The Principle of Justice

Beauchamp & Childress examine several philosophical theories of justice, including egalitarian theories which emphasise "equal access to the goods in life that every rational person values (often invoking material criteria of need and equality) [11]." Beauchamp & Childress propose that "society recognize an enforceable right to a decent minimum of health care within a framework for allocation that incorporates both utilitarian and egalitarian standards [11]." (Utilitarian theories emphasise "a mixture of criteria for the purpose of maximizing public utility") [11].

Rendtorff & Kemp [12] have formulated a European alternative to Beauchamp & Childress' theory using a phenomenological analysis which is based on expressions of the concrete phenomenological reality of the everyday human life world. They state that Beauchamp & Childress have a minimalist conception of the human person which regards autonomy as the only guiding principle. Individuals' capacity for reasoning may be limited or nonexistent. They say that this is not only the case for children, senile people, insane people, etc, but also for normal, intelligent people who feel themselves weak and dependent on others, or who simply do not understand the scientific project they are asked to participate in. Rendtorff & Kemp believe that there are situations where the principle of autonomy does not apply, for instance, unborn life, embryos, the foetus, the human body, etc. [12]. Accordingly, they assert that other supplementary principles must be taken into account, such as respect for dignity, integrity and vulnerability, to protect the human person in biomedicine [12]. Please, find a brief formulation of the principles of Rendtorff & Kemp below:

#### Autonomy

Five important meanings of autonomy can be put forward:

• The capacity for the creation of ideas and goals for life

• The capacity for moral insight

• The capacity for 'Self-legislation' and privacy

• The capacity for rational decision and action without coercion

• The capacity for giving informed consent to medical experiments [12].

#### Dignity

The concept of dignity has two important dimensions. Originally, it expressed an intersubjective recognition of a distinct characteristic or aspect of personality. In that sense it is the quality of being a worthy or honourable person in society. Secondly, dignity becomes a characteristic that every human being has as such, requiring that we must respect our fellow human being as a bearer of rights and duties. In this context dignity signifies a substantial aspect and the intrinsic value of the humanity of the person [12].

#### Integrity

The definition of integrity includes the following moral dimensions:

• Integrity as a created and narrated coherence of life, as a wholeness and completeness of a life story that must not be violated

• Integrity as a personal sphere for experience, creativity and personal self-determination [12].

#### Vulnerability

The temporal and finite quality of all human life indicates that the human condition is fragile. The influence of mortality and destiny on human life cannot be ignored. Vulnerability means that we have to live with morality and take care of the other as a fragile situated subject. Vulnerability is important as the foundation for the notions of care, responsibility and empathy with the other [12].

The theory of Rendtorff & Kemp is an example of a general critique of Beauchamp & Childress' theory. Rendtorff & Kemp's basic ethical principles are promoted in the framework of solidarity and responsibility and take their point of departure in intersubjectivity in contrast to Beauchamp & Childress who base their principlism in a liberal idea of the person in an isolated sense (as their critics would express it). Generally, the critique of Beauchamp & Childress' theory has at least two main complaints: Firstly, Beauchamp & Childress focus too much on individualism, individual rights and the primacy of the individual in the doctor-patient relationship. Secondly, Beauchamp & Childress have a too narrow focus on the self as independent and rationally controlling. The critics question the model of an independent rational will that is inattentive to emotions, communal life and reciprocity. However, in recent years some feminists and care ethicists have sought both to affirm autonomy and to revise individualistic or atomistic conceptions of autonomy through ideas of relational autonomy that centre on the conviction that persons are socially embedded and that agents' identities are formed within the context of social relationships [12,14-17].

If we turn to empirical work, a Dutch study by van Thiel & van Delden [18] shows that respect for autonomy interpreted in a liberal way with a focus on independence and self-determination is too narrow in the context of care in nursing homes. However, van Thiel & van Delden [18] found that caregivers in nursing homes do not prefer a view on good care that is based solely on an ethics of care over a view based on a liberal understanding of the principle of respect for autonomy. Based on the findings, the Dutch researchers formulated a normative multidimensional understanding of the principle, where four moral concepts are relevant, namely protection of freedom, reasonableness, people's choices as part of their life story and the moral element of care as being essential parts of respect for autonomy.

As can be seen above, there is controversy in bioethics about what principles should be used to analyse ethical problems in biomedicine and how to formulate and interpret them in order to reflect the practice of biomedicine. Beauchamp [19] claims, that the efficacy of the four principles of respect for autonomy, beneficence, nonmaleficence and justice can be tested empirically. He does not present any empirical data generated systematically by qualitative research methods to support this position. But he does invite the design of an empirical research project to investigate the question [19]. The Dutch study mentioned above is such an example, it does not simply intent to describe systematically the moral judgements of care givers, it demonstrates how a normative view on respect for autonomy can be formulated by using the method of reflective equilibrium as a normative-empirical model in bioethics [1,18,20]. However, not much research has been done in this field, therefore the overall purpose of the larger research project 'Bioethics in Theory and Practice' was to investigate ethical reasoning in biomedical practice in Denmark empirically having a normative approach [6]. This article presents partial findings of this project by dealing specifically with the principle of respect for autonomy.

### Aim

The aim of this study was to gain insight into how Danish oncology physicians and Danish molecular biologists experience the principle of respect for autonomy in their daily work.

## Methods

The basic approach of the project was phenomenological hermeneutical. This approach was used both for the design of the interview guide and for the interpretation of the data. However, to have a normative approach, the phenomenological hermeneutical method was combined with the moral philosophical method of WRE as a decision procedure in the construction of the interview guide as described in Ebbesen & Pedersen [6].

### Sample

This study is based on 12 semi-structured interviews with three groups of respondents: a group of Danish oncology physicians working in a clinic at a public hospital and two groups of Danish molecular biologists conducting basic research, one group employed at a public university and the other in a private biopharmaceutical company (Table 1). The type of sampling used was random purposeful (random selection to select limited numbers of cases from a larger purposeful sample). The sample size was determined in relation to data saturation. The decisive criterion for sample size is the point where variation ceases; saturation tends to occur when the number of interviews reaches around 15 ± 10 [21,22]. We observed that data saturation was beginning to appear after interviewing 9 respondents (three respondents in each group). The inclusion criteria for this study were that the participants should have an academic degree in medical science or molecular biology and more than five years of working experience, so they have a thorough and profound knowledge of the practice. Those excluded from the study, included people who do not meet the inclusion criteria, do not speak Danish or have not been brought up in Denmark.

**Table 1:** Sample description

**Respondents **(number)	**Respondent group**	**Age **(years)	**Males **(number)	**Females **(number)
4	Danish oncology physicians working in a clinic at a public hospital	45–59	3	1
4	Danish molecular biologists employed at a public university working in a laboratory conducting basic research	31–57	2	2
4	Danish molecular biologists employed in a private biopharmaceutical company working in a laboratory conducting basic research	36–57	1	3

### Interview guide

The ethical reasoning of oncology physicians and molecular biologists was explored by use of semi-structured interviews [22]. The interview guide used consists of 13 main questions (Table 2), each containing a number of sub-questions (the sub-questions are not shown in the table). The single interview lasted for 1 hour and 5 minutes in average and the interview texts were transcribed word-for-word. Please find a detailed description of the theory behind the interview questions in Ebbesen & Pedersen [6].

**Table 2:** Interview guide. Main questions of the interview guide used in the present study of the ethical reasoning of oncology physicians and molecular biologists.

**The interview guide below was used in the present study of the ethical reasoning of oncology physicians and molecular biologists. It consists of 13 main questions, each containing a number of sub-questions (the sub-questions are not presented here)**.
1. Please describe your background
2. Please describe your working day
3. What are the positive/satisfactory aspects of your job?
4. What are the negative/unsatisfactory aspects of your work?
5. In your profession, what makes a person qualified?
6. What are the perspectives of your research?
7. Have you ever been faced with difficult decisions about whether or not to participate in a research project? Or how to treat a patient?
8. Do you feel well-prepared to assess ethical problems about your participation in a research project? Or about what kind of treatment a patient should receive?
9. Presentation of an actual case:
In 2003, it was reported in *Science *that 2 out of 10 patients treated with retroviral mediated gene therapy against the immune system disease SCID-X1 developed leukaemia three years after the treatment. The gene therapy resulted in a functional immune system in 9 out of 10 patients, but 2 patients developed T cell leukaemia caused by insertional mutagenesis. What is your immediate assessment of this case?
10. Presentation of an actual case:
In 2002, the Danish newspaper *Information *reported how an Italian obstetrician had fertilized an infertile woman using the clone of a man. What is your immediate assessment of this case?
11. Presentation of bioethical principles:
Some bioethicists argue that four ethical principles have to be balanced when it comes to assessing bioethical cases: Respect for the patient's autonomy, an obligation to do good (beneficence), an obligation not to harm (nonmaleficence) and just and equal distribution of welfare services. How do you understand these concepts? Are these principles at stake in your practice?
Other bioethicists believe that the principle of respecting the patient's autonomy is too narrow to protect the human person, and that it should be supplemented with the principles of respect for the patient's dignity, vulnerability and integrity. How do you understand these concepts? Are these principles at stake in your practice?
12. Is the amount of time/resources available to you in your daily work sufficient to reflect on ethical issues?
13. Have you been involved in the implementation of concrete initiatives, projects or seminars about ethical issues in your profession?

### Data analysis

The data from the present study were analysed using a phenomenological hermeneutical method for interpreting interview texts inspired by the theory of interpretation presented by Ricoeur as cited in Lindseth & Norberg [4] and Pedersen [23]. In the following the three steps of data analysis are briefly described. For further details please see Lindseth & Norberg [4] and Pedersen [23].

#### Naïve reading

The text is read several times in order to grasp its meaning as a whole. The interpreter tries to read the text with a phenomenological attitude, so as to be open enough to allow the text to speak to him/her. The naïve reading is regarded as a first conjecture and it has to be validated or invalidated by the subsequent structural analysis [4].

#### Structural analysis

According to Ricoeur to understand a text is to follow its movement from what it says to what it talks about [23]. In the structural analysis we move from what the text says to what it talks about, first by describing units of meaning (what is said) and next by identifying and formulating units of significance (what is talked about) and themes (Table 3) [23].

**Table 3:** Example of structural analysis – the movement from what is said to what is talked about, first by describing units of meaning (what is said) and next by formulating units of significance (what is talked about) and themes.

**Respondent group**	**Units of meaning (What is said)**	**Units of significance (What is talked about)**	**Themes**
**Molecular biologist employed at the university (MBU, Q1)^a^**	You must inform them of their options and then respect their decision.	**Inform patients and respect their decision**	**Respect for autonomy/Informed consent** • **Respect decision** • **External constraints**
**Molecular biologist employed in a private biopharmaceutical company (MBP, Q2)^b^**	... if you were a seriously ill or terminally ill patient, I think I would accept just about any treatment, because you would accept the risk involved.	**Very sick patients are constraint by the circumstances to a certain choice**	**Respect for autonomy/Informed consent** • **External constraints** • **Vulnerability** • **Fragility**
**Oncology physician working in the clinic (OPC, Q3)^c^**	... try to determine what is wrong with the patient, what are our options, what are the patient's wishes, ideas, and then we have to reach some kind of mutual understanding, frame of reference and take it from there ... and how can we deal with this in respect of that.	**Medical prognosis** **Risk-benefit analysis** **Patient's wishes and ideas** **Mutual understanding** **Respect**	**Medical prognosis** • **Risk-benefit analysis** **Respect for autonomy/Informed consent** • **Patient's wishes and ideas** • **Mutual understanding** • **Respect**
**Oncology physician working in the clinic (OPC, Q4)**	... patients are very different and you must adjust to their level as best you can and try to find out what kind of language to speak and to sense if they have understood what you have told them, and maybe repeat it...	**Positive obligation to adjust to the level of the patient** **Information** **Understanding**	**Respect for autonomy/Informed consent** • **Disclosing information** • **Probing for understanding**

^a^(MBU, Q1): **M**olecular **B**iologist employed at a public **U**niversity, **Q**uotation **1**.

^b^(MBP, Q2): **M**olecular **B**iologist employed in a **P**rivate biopharmaceutical company, **Q**uotation **2**.

^c^(OPC, Q3): **O**ncology **P**hysician working in the **C**linic at a public hospital, **Q**uotation **3**.

First, the whole text is read and divided into units of meaning (what is said). These units of meaning can be part of a sentence, a sentence or a paragraph. Secondly, the units of meaning are reflected on in relation to the naïve understanding. Then the units of meaning are sorted and condensed and units of significance are formulated (what is talked about). Next, units of significance are condensed even more and themes are formulated. A theme is a thread of meaning that penetrates text parts. A theme identifies an essential meaning of lived experience; these themes are formulated as condensed descriptions and abstract concepts [4,23].

During the structural analysis the text is viewed as objectively as possible by decontextualising the units of meaning from the text as a whole, thus the text parts are considered as independently as possible from their context in the text [4,23].

The themes are reflected on against the background of the naïve understanding to see whether the themes validate or invalidate the naïve understanding. If the structural analysis invalidates the naïve understanding, the whole text is read again and a new naïve understanding is formulated and checked by a new structural analysis. This process of comparing the naïve reading and the structural analysis is repeated until the naïve understanding is validated through the structural analysis [4,23].

#### Critical interpretation

The themes are reflected on in relation to the literature. The text is read again as a whole with the naïve understanding and the validated themes in mind and with an as open mind as possible. However, according to Lindseth & Norberg [4] we interpret in relation to our pre-understanding and we cannot free ourselves from this pre-understanding. This is in line with Gadamer who thinks that the hermeneutic mode of interpreting meaning is not presupposition-less, as the phenomenological description is. The interpreter of texts cannot 'jump outside' the tradition of understanding he/she lives in [24,25]. The interpreter should, however, attempt to make his/her presuppositions or foreknowledge explicit [24]. The foreknowledge in the present study for instance includes the bioethical principles of Beauchamp & Childress [11] and Rendtorff & Kemp [12]. According to Lindseth & Norberg [4], one can find literature that may be appropriate for helping to revise, widen and deepen our understanding of the text. This is where existing bioethical theory for data interpretation comes in. The bioethical principles of respect for autonomy, beneficence, nonmaleficence and justice of Beauchamp & Childress [11] and the principles of respect for autonomy, dignity, integrity and vulnerability of Rendtorff & Kemp [12] can be used to structure the comprehensive understanding of the text, present alternative views and maybe revise the structure already made. The reading and interpretation of interview texts should be performed as open-mindedly as possible to insure that the different interpretations in the principles of Beauchamp & Childress [11] and Rendtorff & Kemp [12] may both contribute to an understanding of the lived experience of the interviewees. However, since this was a phenomenological hermeneutical study we did not force the literature perspective on the interview text but let the literature illuminate the interview text and the interview text illuminate the literature [4,23].

## Results

From the structural analysis a number of themes emerged, these themes are explored in details below.

### Informed consent, external constraints and vulnerability

In this study we see examples of how molecular biologists experience that informed consent can be influenced by external constraint. Below we present selected quotations showing how the themes and sub-themes of informed consent, external constraints and vulnerability are reflected in the interviews.

For instance, in MBU, Q1, Table 3, a molecular biologist employed at the university tells that patients must be informed of their options regarding treatment or trials and that their decision regarding these issues should be respected. This quotation indicates that informed consent should be respected without external constraints. However, in MBP, Q2, Table 3, a molecular biologist employed in a private biopharmaceutical company stresses that very sick patients would accept any treatment, they would accept the risks involved, they are vulnerable and constraint by the circumstances to a certain choice. MBU, Q5, presented below, illuminates the same issues telling that patients and research subjects should decide themselves, but that very sick patients are constraint by the circumstances to a certain choice.

MBU, Q5:

... people make their own choices; if you inform people of the risks, they must make the decision themselves. The problem is if they feel they are forced into it. Some may feel this way; it depends on the person.

### Respect for autonomy based on the patient's wishes and ideas, information and understanding

This study shows examples of how oncology physicians experience that informed consent or refusal is based on the patient's wishes and ideas, information and understanding. For instance OPC, Q3, Table 3, tells that the physician in dialogue with the patient performs a medical prognosis or risk-benefit analysis based on the patient's wishes and ideas, mutual understanding and respect. Furthermore, OPC, Q4, Table 3, stresses that the physician has a positive obligation to adjust to the level of the patient when disclosing information making sure that the patient understands. OPC, Q6, below, stresses that the tasks of the physician are 1) To disclose information so that the patient can make informed consent and 2) To respect this informed consent. However, OPC, Q6 also reflects the principle of respect for autonomy as a negative obligation, since the physician must respect the decision even though it is not the one he/she has recommended.

OPC, Q6:

My task is to ensure as well as possible that they know ... receive information on what *we *can offer and what options are available to them in their situation. And that the information is communicated in such a way that it forms the basis of their decision-making. If they then decide something else, then that is that.

### The principle of respect for autonomy does not apply

Oncology physicians experience that there are situations, where the principle of respect for autonomy does not apply. OPC, Q7, below, describes such a situation.

OPC, Q7:

... when we have a protocol like that – there is the inclusion criteria ... the patient may meet all the criteria, but when I sit in front of the patient, I think to myself: this just does not work. This patient is in some sort of crisis or situation in which it is not fair to ask them to make this kind of decision. And then I can choose to say to myself that it is not fair. Then we give them the standard treatment ... once in a while I decide that they are not capable of making these decisions themselves. It is not fair to place the strain and stress of having to make such a decision on them – because it *is *a strain.

OPC, Q7 stresses that the physician's decision about treatment depends on the physical and psychological condition of the patient. Furthermore, the quotation tells that the physician avoids asking the patient to make a decision or not if the patient is in a difficult situation, since it is not fair or just under these circumstances to place stress on the patient.

### Family and physician autonomy

OPC, Q8:

... I prefer that the important decisions are made in consultation with the family – and that our decisions are accepted by the family. Because they are the ones who must live on and feel that things have been done in a decent way. So it is important for me that the family backs up the decision. It is preferable if we can agree on the decision, but if the family and I disagree strongly, we may be forced to make a decision that goes against the family. It is still in the family's interest ...

OPC, Q8, above, describes how the physician prefers making important decisions in consultation with the family. Furthermore, the quotation tells that the physician prefers that the family supports and accepts these decisions. However, if the physician and the family disagree, the autonomy of the physician overrides the autonomy of the family having the well-being of the patient in focus.

### Falls beliefs, lack of understanding and acceptance

OPC, Q9:

... an increasing number of patient complaints that ... may reflect the fact that we have become less competent – I am not sure, but it may reflect the fact that it becomes increasingly difficult for people and patients to accept that not everything can be cured. Yes, everything can be treated and everything can be diagnosed, and everything is ... I mean, if you make a scan, the answer is very precise – it is black and white: you are either ill or healthy. The fact that there may be shades of grey and the fact that results must be interpreted and so on is likely to become increasingly difficult for patients to understand.

OPC, Q9 tells that the number of patient complaints is rising. Patients believe that every disease can be cured and they do not accept the limits of treatment. Furthermore, the quotation tells that it is difficult for patients to understand that results need to be interpreted and that samples do not always give clear answers. This reflects that patients have falls beliefs, do not understand the information provided and therefore do not accept the situation.

OPC, Q10, below, indicates a shift in the action pattern of patients. Previously, the patient told the physician when to stop treatment if it seemed hopeless, these days oncology physicians have to set the limits for treatment themselves.

OPC, Q10:

When we reach a point where my professional experience tells me that more chemotherapy will not be meaningful, it will only cause side-effects and it will not do any good. It is quite often me who suggests a termination of the therapy – rather that the patient telling me he or she wants to stop. There has been a clear shift in the patients' action pattern over the last many years.

## Discussion

In the structural analysis above, we did not see any difference in the experience of the two groups of molecular biologists, the one employed at a public university and the other in a private biopharmaceutical company. Therefore, in the critical interpretation presented below, we consider these two groups as one large group of molecular biologists.

### The application of the principle of respect for autonomy

This study shows examples of how Rendtorff & Kemp's bioethical theory is reflected in the interviews of Danish oncology physicians and Danish molecular biologists. When interpreting the interview texts we see *similarities *between the group of molecular biologists and oncology physicians. We see the general picture that two of the five important meanings of autonomy that Rendtorff & Kemp put forward are reflected: 1) The capacity for rational decision and action without coercion and 2) The capacity for giving informed consent to medical experiments [12]. These concepts are reflected in MBU, Q1, which tells that patients must be informed and that their decision should be respected. However, at the same time MBP, Q2 stresses that very sick patients are constraint by the circumstances to a certain choice. This reflects Rendtorff and Kemp's concept of vulnerability, which means that we have to take care of the other person as a fragile situated subject [12].

However, we also see *differences *between the groups of molecular biologists and oncology physicians. In contrast to molecular biologists, oncology physicians experience that informed consent or refusal to treatment or trials are based on the patient's wishes and ideas, information and understanding. OPC, Q3 tells that the physician in dialogue with the patient performs a medical prognosis or risk-benefit analysis based on the patient's wishes and ideas, mutual understanding and respect. This reflects another of the five important meanings of autonomy that Rendtorff & Kemp put forward, namely the capacity for the creation of ideas and goals for life [12]. At the same time OPC, Q3 reflects the concepts of dignity and integrity of the patient. According to Rendtorff & Kemp, the concept of dignity tells that we must respect our fellow human being as a bearer of rights and duties and the concept of integrity says that integrity as a created and narrated coherence of life and as a personal sphere for experience, creativity and personal self-determination must not be violated. OPC, Q4 stresses that the physician has a positive obligation to adjust to the level of the patient when disclosing information making sure that the patient understands. However, we do not see this positive obligation of respect for autonomy reflected in the bioethical theory of Rendtorff & Kemp. Furthermore, the theory of Rendtorff & Kemp does not reflect the positive obligation of the physician to perform a medical prognosis in dialogue with the patient based on the patient's wishes, ideas, mutual understanding and respect.

This study also shows examples of how Beauchamp & Childress' bioethical theory is reflected in the interviews of Danish oncology physicians and Danish molecular biologists. When interpreting the interview texts in relation to Beauchamp & Childress' theory, we see that the groups of molecular biologists and oncology physicians both experience the principle of respect for autonomy as a negative obligation, which says that autonomous actions should not be subjected to controlling constraints by others [11]. This is for instance seen in MBU, Q1, where a molecular biologist tells that patients must be informed of their options regarding treatment or trials and that their decision should be respected. This quotation indicates that informed consent should be respected without external constraints. However, in contrast to molecular biologists, oncology physicians also experience the principle of respect for autonomy as a positive obligation in line with Beauchamp & Childress' theory, which requires respectful treatment in disclosing information, probing for and ensuring understanding, and voluntariness and fostering autonomous decision-making [11]. This is seen in OPC, Q3 stressing that the physician in dialogue with the patient performs a medical prognosis or risk-benefit analysis based on the patient's wishes and ideas, mutual understanding and respect. The principle of respect for autonomy as a positive obligation is also reflected in OPC, Q4, which stresses that the physician has a positive obligation to adjust to the level of the patient when providing information making sure that the patient understands. OPC, Q6 expresses the principle of respect for autonomy as both a negative and as a positive obligation. It tells that the task of the physician is 1) To disclose information so that the patient can make informed consent and 2) To respect this informed consent. Inspired by Beauchamp & Childress this quotation reflects respect for autonomy as a positive obligation, since the task of the physician is to disclose information, probing for and ensuring understanding and fostering autonomous decision making. However, OPC, Q6 also reflects the principle of respect for autonomy as a negative obligation, since the physician must respect the decision even though it is not the one he/she has recommended.

### The principle of respect for autonomy does not apply – the patient is not competent

OPC, Q7 stresses that the physician's decision about treatment depends on the physical and psychological condition of the patient. Furthermore, the quotation tells that the physician avoids asking the patient to make a decision or not if the patient is in a difficult situation, since it is not fair or just under these circumstances to place stress on the patient. Inspired by Beauchamp & Childress, this quotation describes a situation where the principle of respect for autonomy does not apply because of the physical and psychological condition of the patient. The patient is not competent, i.e. the patient is not able to make autonomous decisions. Beauchamp & Childress define competence to decide about treatment or about participation in research the following way: "Patients or subjects are competent to make a decision if they have the capacity to understand the material information, to make a judgement about the information in light of their values, to intend a certain outcome, and to communicate freely their wishes to care givers or investigators [11]."

However, OPC, Q7 can also be interpreted in light of Rendtorff & Kemps theory. According to Rendtorff & Kemp, individuals' capacity for reasoning may be limited or nonexistent. They say that this is not only the case for children, senile people, insane people, etc, but also for normal, intelligent people who feel themselves weak and dependent on others, or who simply do not understand the scientific project they are asked to participate in. Rendtorff & Kemp believe that there are situations where the principle of autonomy does not apply. Accordingly, they assert that other supplementary principles must be taken into account, such as respect for dignity, integrity and vulnerability, to protect the human person in biomedicine [12]. The situation described in OPC, Q7 might be such a situation.

Alternatively, OPC, Q7 can also be considered as a justice consideration: It is not fair or just under the circumstances to ask the patient in that specific situation to participate in research. Basically, OPC, Q7 is about inclusion criteria. However, in light of Beauchamp & Childress' theory the case can also be viewed from the point of a balance of the principles of beneficence and nonmaleficence. According to the physician, it is too large a burden to put on the patient in that specific situation to ask him/her to participate in research.

## Conclusion

If we turn to the themes of the structural analysis, generally we see that three of the five important meanings of autonomy that Rendtorff & Kemp put forward are reflected in the interviews: 1) The capacity for rational decision and action without coercion, 2) The capacity for giving informed consent to medical experiments and 3) The capacity for the creation of ideas and goals for life [12]. Oncology physicians and molecular biologists tend to stress rational decision making, action without coercion and informed consent. However, in contrast to molecular biologists, oncology physicians stress the importance of the capacity of the patient for the creation of ideas and goals for life. Rendtorff & Kemp's concepts of dignity and integrity are also only reflected in the interviews of oncology physicians. According to Rendtorff & Kemp the concept of dignity tells that we must respect our fellow human being as a bearer of rights and duties and the concept of integrity says that integrity as a created and narrated coherence of life and integrity as a personal sphere for experience, creativity and personal self-determination must not be violated.

When we interpret the themes of the structural analysis in light of Beauchamp & Childress' theory, we see that oncology physicians and molecular biologists stress the principle of respect for autonomy of Beauchamp & Childress as a negative obligation, which says that autonomous actions should not be subjected to controlling constraints by others [11]. However, in contrast to molecular biologists, oncology physicians stress the principle of respect for autonomy as a positive obligation, which requires respectful treatment in disclosing information, probing for and ensuring understanding, and voluntariness and fostering autonomous decision-making [11]. The reason why molecular biologists and oncology physicians perceive or experience the principle of respect for the patient or the research subject in different ways might be because of the fact that molecular biologists investigate DNA and cells in cultures and thereby do not face the patient directly, while oncology physicians do work with human beings in a doctor-patient relationship.

To conclude, this study shows that crucial parts of the bioethical theory of Rendtorff & Kemp are reflected in the interviews. However, the positive obligations of the physician which are illuminated in the interviews are not part of the theory. The study also shows that essential parts of Beauchamp & Childress' theory are reflected in the interviews. But importantly, Beauchamp & Childress' theory does not specifically stress the constrained situation of the patient such as Rendtorff & Kemp's theory does. One can argue that the constrained situation or the vulnerability of the patient is a condition and can not be considered as an ethical principle, therefore it is not explicitly included in Beauchamp & Childress' theory. When the patient is constraint or vulnerable, the principles of beneficence and nonmaleficence step in and protect the patient.

To conclude, this study shows that essential parts of the theories of Beauchamp & Childress and Rendtorff & Kemp are reflected in the daily work of Danish oncology physicians and Danish molecular biologists, but the study also explores dimensions where the theories can be developed further to be concordant with biomedical practice. The hope is that this study enhances the understanding of the principle of respect for autonomy and the way it is practiced.

## Competing interests

The author(s) declare that they have no competing interests.

## Authors' contributions

ME and BDP designed the study. ME carried out the interviews and the analysis, wrote the manuscript and completed and approved to the final manuscript. BDP participated in the analysis and choice of methods and critically approved to the final manuscript.

## Appendix

Note 1: Following Tom L. Beauchamp & James F. Childress, we understand the term bioethical principles as "general norms that leave considerable room for judgment in many cases. They do not act as precise action guides that inform us in each circumstance how to act in the way that more detailed rules and judgments do [11]."

Note 2: The notion of 'coherence' is not well defined. Philosophers agree that coherence is not only characterised by consistency [26,27]. According to the Danish philosopher, Klemens Kappel [27], coherence is characterised by consistency, systematicity (a belief set should contain explanatory relations), generality (a belief set should contain general beliefs that cover a larger area rather than a smaller one), and simplicity (general explanatory beliefs should be few and simple rather than many and complex).

Note 3: Our note. According to Rawls, a competent judge possesses the following characteristics: Intelligence and knowledge, intellectual virtues of reasonableness, an open mind, and sympathetic knowledge of those human interests which give rise to the need to make a moral decision [10].

## Pre-publication history

The pre-publication history for this paper can be accessed here:


